# Combined Diffusion Tensor Imaging and Quantitative Susceptibility Mapping Discern Discrete Facets of White Matter Pathology Post-injury in the Rodent Brain

**DOI:** 10.3389/fneur.2020.00153

**Published:** 2020-03-06

**Authors:** Neha Soni, Viktor Vegh, Xuan Vinh To, Abdalla Z. Mohamed, Karin Borges, Fatima A. Nasrallah

**Affiliations:** ^1^Queensland Brain Institute, The University of Queensland, Brisbane, QLD, Australia; ^2^Center for Advanced Imaging, The University of Queensland, Brisbane, QLD, Australia; ^3^Faculty of Medicine, School of Biomedical Sciences, The University of Queensland, Brisbane, QLD, Australia

**Keywords:** traumatic brain injury, diffusion tensor imaging, track-based spatial statistics, quantitative susceptibility mapping, myelin, gliosis, contralateral

## Abstract

Early loss of white matter microstructure integrity is a significant cause of long-term neurological disorders following traumatic brain injury (TBI). White matter abnormalities typically involve axonal loss and demyelination. *In-vivo* imaging tools to detect and differentiate such microstructural changes are not well-explored. This work utilizes the conjoint potential offered by advanced magnetic resonance imaging techniques, including quantitative susceptibility mapping (QSM) and diffusion tensor imaging (DTI), to discern the underlying white matter pathology at specific time points (5 h, 1, 3, 7, 14, and 30 days) post-injury in the controlled cortical impact mouse model. A total of 42 animals were randomized into six TBI groups (*n* = 6 per group) and one sham group (*n* = 6). Histopathology was performed to validate *in-vivo* findings by performing myelin basic protein (MBP) and glial fibrillary acidic protein (GFAP) immunostaining for the assessment of changes to myelin and astrocytes. After 5 h of injury radial diffusivity (RD) was increased in white matter without a significant change in axial diffusivity (AxD) and susceptibility values. After 1 day post-injury RD was decreased. AxD and susceptibility changes were seen after 3 days post-injury. Susceptibility increases in white matter were observed in both ipsilateral and contralateral regions and persisted for 30 days. In histology, an increase in GFAP immunoreactivity was observed after 3 days post-injury and remained high for 30 days in both ipsilateral and contralateral white matter regions. A loss in MBP signal was noted after 3 days post-injury that continued up to 30 days. In conclusion, these results demonstrate the complementary ability of DTI and QSM in discerning the micro-pathological processes triggered following TBI. While DTI revealed acute and focal white matter changes, QSM mirrored the temporal demyelination in the white matter tracts and diffuse regions at the chronic state.

## Introduction

More than 70% of brain traumas encompass axonal injury (TAI) (1). TAI is a progressive event that gradually evolves from focal axonal impairments to delayed and diffused axonal damage (2). Demyelination is one of the major factors of traumatic axonal injury and is evident by fragmentation and loss of myelin sheaths (1, 3–5). Research supports that both axonal degeneration and myelin degradation have a significant impact on the long-term consequences of traumatic brain injury (TBI) and are important biomarkers to be diagnosed at an early stage (1, 6). Currently, computerized tomography imaging and assessment of neurological dysfunction are the only clinical tools to determine the neuropathies associated with white matter damage, and these approaches only provide limited information (7–9). MRI-based *in vivo* biomarkers, which are specific, sensitive, and non-invasive, and can detect axonal damage and demyelination reliably, still require further validation before they can reliably be used in the assessment of white matter damage caused by TBI.

There is extensive evidence on the potential of DTI to detect the microstructural alterations in white matter after brain injury (10, 11). DTI is an established MRI technique, which relies on water diffusion in tissue from which tissue microstructure characteristics are inferred (12, 13). White matter-associated DTI-derived parameters include fractional anisotropy (FA), a measure reflecting the extent of tissue anisotropy within an MRI voxel, axial diffusivity (AxD), water diffusivity in the principal direction, i.e., oriented along white matter fiber bundles, radial diffusivity (RD), water diffusivity perpendicular to principal direction, and mean diffusivity (MD), the overall diffusivity within the MRI voxel (14). Fractional anisotropy has been widely used clinically and preclinically for the measurement of microstructural changes in the white matter following different types of brain injuries (11, 15, 16). However, the measurements have been shown to lack specificity with respect to pathology (17, 18). Other DTI-derived measures, namely axial and radial diffusivity, have been shown to provide more robust information on axon and myelin integrity (3, 19). Changes in AxD have been linked with axonal damage and changes in RD reflect changes in myelination, or myelin integrity (19–21). AxD and RD together with FA have been suggested to be potential biomarkers for the detection and discrimination of axon and myelin degeneration (22, 23). Arfanakis et al. showed that in mild TBI patients at 1-day post-injury, reduction in FA and AxD, along with an increase in RD corresponded to diffuse axonal injury. This was concluded based on previously published histological findings on axonal swelling, damage, and recovery (24). In a different TBI study, a proportional increase in AxD and RD without a significant change in FA at the acute stage (within 1–3 days) was followed by a disproportionate increase in AxD and RD in conjunction with a decrease in FA at the chronic stage (after 7 months) in patients (25). Early post-injury changes in RD were suggested to be a biomarker of oedema and demyelination (25). Song et al. specifically explored the potential of AxD and RD as biomarkers of axonal damage and myelin degeneration in a mouse model of retinal ischemia (3). They reported a decrease in both AxD and RD on day 3 followed by increased RD on day 5, which correlated with their histological findings of axonal degeneration on day 3 and myelin damage on day 5. In other rodent studies, an increase in RD with either reduced or no change in AxD was reported early after injury, again linked with myelin and axonal damage based on histology (26, 27). While white matter microstructural alterations affect DTI measures, the metrics appear to be non-specific encompassing an array of pathological changes occurring after TBI (18, 28). Notably, in the case of more severe injuries, AxD and RD may provide insufficient information on the specific white matter changes taking place (29, 30). Therefore, supporting detail needs to be derived from additional data, such as magnetic susceptibility.

Quantitative susceptibility mapping (QSM) is a well-established MRI method used to quantify changes in the magnetic susceptibility of image voxels (31). QSM is a post-processing technique which derives information from the gradient recalled echo MRI signal phase (i.e., phase image), and the signal magnitude (i.e., magnitude image), which may serve a number of purposes, including the creation of phase masks and structural priors within the image. The contrast in images generated using QSM has been associated with changes in the magnetic properties of the bulk tissue consequential of microscale changes in, for example, iron (haem/ non-haem), calcium, and myelin (32–35). In the white matter, a decrease in phase image contrast is indicative of macro- and micro-hemorrhages or increased haem and non-haem iron content in tissue (36, 37). In addition, QSM can likely be used to monitor myelin levels and thereby demyelination in tissue at the voxel scale as the magnetic properties of myelin are vastly different to that of surrounding tissue constituents (37, 38).

As the magnetic properties of tissue can be affected by both the cellular composition and arrangement of fiber bundles (39, 40), combining DTI with QSM to detect axonal damage and demyelination can potentially lead to new insights and more robust identification of white matter pathology post-injury. To date, QSM has provided additional insight into white matter pathologies in multiple sclerosis and Alzheimer's disease (36, 41–43). Yet there has been a lag in the acceptance of the method for the analysis of traumatic axonal injury. Recently, Koch et al. reported increased QSM values in concussion patients in white and gray matter within 24 h post-injury that persisted up to 8 days and normalized after 6 months (44). Pathophysiological reasons for the changes were not provided. In a study on hockey players, Weber et al. focused on AxD changes as a measure of axonal damage and QSM and RD for myelination (45). They did not find any differences between the methods, which may be explained by the low severity of the injury. Li et al. combined QSM with DTI metrics in a rat controlled cortical impact (CCI) model (34). They observed increased susceptibility and RD in the demyelinating regions of the corpus callosum close to the injury site and reported QSM to be potentially more sensitive and specific to detect myelin changes near the injury site.

QSM and DTI are advanced imaging techniques that have shown great potential in detecting microstructural processes in the brain. In view of the complex pathological processes triggered following TBI and the challenges associated with their detection, we hypothesize that combined QSM and DTI measures, i.e., AD and RD, have the potential to discern different aspects of the microstructural pathological changes induced by TBI at early and late stages.

## Materials and Methods

The study was approved by the Animal Research Ethics Committee (AEC) of the University of Queensland (AEC number: QBI/SCMB/036/16/MAIC). A cross-sectional study was performed, first to minimize multiple exposures to isoflurane, reported to have a neuroprotective effect in TBI (46, 47), and to collate histological evidence for multiple time points.

### Animals and Injury Model

Young adult 5 week old CD1male mice (28–30 gm, *n* = 42) were purchased from the animal resource center (ARC, Western Australia). The reasons for choosing males was the known neuroprotective effect of estrogen (48). After 1 week of acclimatization, animals were randomly divided into two groups: (1) the TBI group (*n* = 6, per time point, i.e., 5 h, day 1, 3, 7, 14, and 30 post-injury) and (2) the sham group (one mouse from each time point was combined into one control group (*n* = 6).

The CCI injury model was used for inducing brain injury. Animals were anesthetized with isoflurane (1.5–1.8%) in a mixture of compressed air and oxygen (1:0.8) and fixed on the stereotactic frame. A craniotomy was performed on the left side with dura intact in both TBI and sham groups. Only the TBI groups underwent controlled cortical impact using the CCI device (Precision system and instrumentation PSI) equipped with a piston tip of diameter = 3 mm to create an injury with an impact velocity = 3.5 m/sec, depth = 1 mm, and contact time = 400 ms. The bone flap from the cranial window was placed back to the craniotomy region in the TBI and sham groups, and the skin was sutured. After recovery from the anesthesia, animals were kept in cages with water gel and wet food.

### *In vivo* Data Acquisition

All animals were scanned on a 9.4T Bruker Advance III spectrometer (Bruker Biospec, Germany) equipped with a surface cryoprobe coil (Bruker, Germany). Animals were again anesthetized with 1.5–1.8% isoflurane (chosen to achieve a respiratory rate of 65–75 bpm) with the head straight fixed horizontally onto the scanner bed using a bite bar and ear bars. Temperature and respiration were monitored using an animal monitoring system (SA Instruments, Melbourne, VIC, Australia). Body temperature was maintained at 36.5–37°C using warm water circulation through the bed.

Magnetic field shimming was adjusted for each mouse by using the B0 field map shimming function in a voxel covering the whole brain. The area of injury was included in the voxel for the field map shimming to reduce field distortion as much as possible. Three-dimensional (3D) T2- weighted data were acquired axially using rapid acquisition with a relaxation enhancement (RARE) sequence with repetition time (TR) = 6,800 ms, echo time (TE) = 39 ms, averages = 2, slice thickness = 0.3 mm, field-of-view (FoV) = 19.2 mm × 19.2 mm × 16.5 mm, matrix size = 192 × 192 × 55, scan time = 5 m 26 s, and 400 ms. Diffusion tensor imaging (DTI) data was acquired using an axial gradient echo planar sequence with TR = 10,000 ms, TE = 28 ms, number of slices = 54, slice thickness = 0.25 mm, FoV = 19.2 mm × 12.04 mm × 13.5 mm, matrix size = 128 × 86 × 54, 4 *b* = 0 s/mm^2^, non-collinear directions = 30 each with two *b* = 750 s/mm^2^, and 2,000 s/mm^2^. QSM data were acquired using a 3D multi-echo spoiled gradient recalled echo (GRE) sequence without flow compensation: TR = 50 ms, minTE/ΔTE/maxTE = 3.5/4.74/27.19 ms, echos = 6, averages = 1, FoV = 25.6 mm × 16.0 mm × 12.8 mm, matrix= 256 × 160 × 128, isotropic resolution = 100 μm^3^, spectral width = 64.10256 kHz, bandwidth = 1.4 kHz, flip angle = 15°, scan time = 17 min 4 s.

### Data Processing and Analysis

#### T2-Weighted, DTI, and Track Based Spatial Statistics (TBSS) Analysis, Tractography

Collected T2-weighted data were used to generate a study-specific template for registering the QSM and DTI data using FSL, FMRIB Software Library version 5.0.9 (49, 50). Automatic skull stripping function of FSL, FSL-BET (BET; https://fsl.fmrib.ox.ac.uk/fsl/fslwiki/BET) (49, 50) was used to extract the brain from the skull with minor manual adjustments. Macroscale field inhomogeneity correction was performed on all datasets using the N4BiasFieldCorrection function of the advanced normalization tools (ANTs Version: 2.1.0-gGIT-N). The skull-stripped and inhomogeneity corrected individual T2-weighted images were registered to a common standard Australian Mouse Brain Mapping Consortium (AMBMC) template (http://imaging.org.au/AMBMC/Model) (51) using FMRIB's registration tools (FLIRT and FNIRT). All individual T2-weighted data to AMBMC registered images/group were averaged (*n* = 6 per group) to create a final study-specific template for each time point.

**Figure 1:**
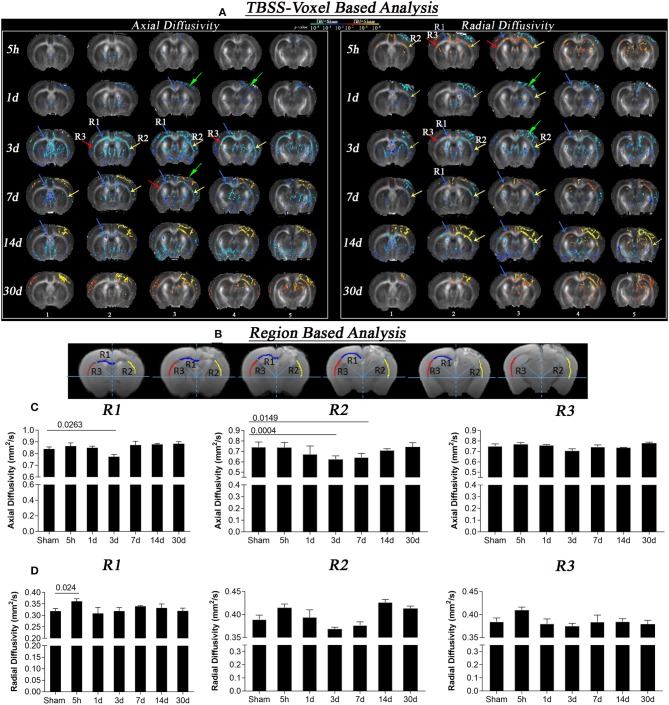
Axial and radial diffusivity changes at different times after CCI (5 h, 1 day, 3, 7, 14, and 30 days). **(A)** Whole brain TBSS voxel-based statistical difference maps for axial and radial diffusion, representing differences between TBI and sham groups at different time intervals post-injury Intergroup difference maps for axial (left) and radial diffusivity (right) between sham and TBI at different time points (red and blue color) overlaid on the mean FA template. Red color shows voxels with significantly higher DTI indices (*z* = 1.96; *p* = 0.05), and blue color indicates voxels with significantly lower AxD and RD values (*z* = 1.96; *p* = 0.05). Maximum changes in AxD were evident on day 3, where reduction was seen that persisted for 14 days. In RD maps, increase in diffusion at 5 h followed by a decrease on day 3 further leads to increase after 7, 14, and 30 days post-injury was seen. Sections 1 to 5 refers to the interaural regions 3.5, 3, 2.5, 2, and 1.5 mm as per AMBMC mouse brain atlas (51). **(B)** Representing regions of interest (region 1 (R1), region 2 (R2), and region 3 (R3) chosen on the basis of voxel based results. **(C)** Graphs representing changes in axial diffusivity in R1, R2, and R3. A significant decrease in AxD values was noted in day 3 TBI group as compare to sham group in R1 and R2 that persisted for day 7 post injury in R2. **(D)** Graphs representing changes in radial diffusivity in R1, R2, and R3. A significant increase in RD values was noted after 5 h of injury as compared to sham group in R1. Non-significant reductions in RD in R2 were noted in R2 followed by non-significant increase at day 14 and 30 post injury. Values expressed as mean ± SEM and non-parametric student *t*-tests were performed; corrected *p*-value were reported.

DTI data sets were additionally corrected for motion using FSL-MCFLIRT. The FSL Diffusion Toolkit (DTI-FIT) was used to calculate the voxel-level diffusion tensor from which maps of FA, MD, AxD, and RD were generated. Using the *b* = 0, DTI data for each mouse were registered linearly using FLIRT to the T2-weighted image template, and the transformation matrix was applied to each DTI metric map, after which the T2-to-T2 template transformation matrix was used to convert DTI metrics into the study-specific template space. TBSS analysis was performed using the standard pipeline implemented in FSL, FMRIB (49, 50). All registered FA maps for the subjects were averaged to generate a mean FA map. The mean FA map was further used to generate a skeletonised mean FA map. The skeletonised mean FA map was used to create a distance map which was then used along with FA threshold 0.2 and the anterior commissure as the reference to generate the TBSS skeleton for the individual FA maps and subsequently for all other individual metrics (AxD, RD, and MD skeletonised). The T_2_-weighted study-specific template was used as an underlay to represent the diffusion changes in Figure 1, where sections 1–5 refer to the interaural regions 3.5, 3, 2.5, 2, and 1.5 mm as per the AMBMC mouse brain atlas (51).

Fiber-tracking was carried out using MRtrix 3.0 (Brain Research Institute, Melbourne, Australia, http://www.brain.org.au/software/) software (52) using motion and inhomogeneity corrected individual DTI datasets. The white matter response function was calculated to estimate unique single fiber white matter for each individual, a requirement for spherical deconvolution. The constrained spherical deconvolution algorithm (53) was employed along with the response function and whole-brain mask to be able to model multiple fiber orientation distributions (FOD) in each voxel (52, 54, 55). Whole-brain tractography images were generated based on FOD maps and whole-brain masks using the tckgen command involving the following parameters: seeds random per voxel 20, max length = 20, cut off = 0.1. Furthermore, corpus callosum (CC) tracks were generated using the same FOD image, using CC seed masks where the number of seeds = 10,000, maximum attempts per seed = 10,000, and seed direction = [1, 0, 0].

Based on the DTI TBSS voxel-wise statistical difference maps between the TBI and the sham group, three regions of interest involving middle corpus-callosum [region 1 (R1)], corpus-callosum-external capsule ipsilateral to injury [region 2 (R2)], and corpus-callosum-external capsule contralateral to injury [region 3 (R3)] of the white matter were selected and manually segmented on the individual T2-weighted data using the two-dimensional manual tracing tool fsleyes (FSL). To keep the masks consistent among different animals, the AMBMC mouse brain atlas and study-specific template were used as a reference. All slices between the interaural regions 3.5–1.5 were covered in the ROI mask. As all the T2-weighted images were registered in the common space (T2-weighted study-specific template), specific XY coordinates/slice were used to draw the regional masks for each slice. For each individual, 222 voxels (~10–12 voxels per slice; total slices 10) were included in R1, and 275 voxels (22–23 voxels per slice; total slices 12) in R2 and R3.To apply these ROI-masks on diffusion data, DTI images were first registered to T2-weighted data to generate the DTI to T2-weighted matrix using flirt, which was then used to register T2-weighted data to the DTI data. All ROI masks were registered likewise. All registered ROI masks were then used to extract specific white matter axial and radial diffusivity values. DTI images from the interaural regions 3.5, 3, 2.5, 2, and 1.5 mm are shown in the Figure 1 (51).

#### Quantitative Susceptibility Mapping

GRE-MRI magnitude and phase images were reoriented followed by skull stripping. The automatic 3D pulse-coupled neural networks (PCNN) tool (56) was used to generate brain masks from magnitude images, which were corrected manually. Background field effects in phase images were removed using homodyne filtering, resulting in tissue phase images. At each echo time, tissue phases were converted to frequency shifts (in Hz) by dividing each tissue phase value by the relevant echo time multiplied by 2π. Tissue phases were converted to susceptibility maps (QSM in ppb) using the iLSQR function available in STI Suite Version 3.2 available in MATLAB. Frequency shift (fs) and quantitative susceptibility values at each voxel were referenced to the whole brain mean value. Due to the impact of inhomogeneity of the injury on the magnetic field of the scanner, and therefore, the GRE-MRI signal, the shortest echo time images were found to produce the clearest results in terms of frequency shift and susceptibility mapping. This is because macroscale susceptibility effects increase with echo time and become difficult to remove using the background field filtering step.

The individuals' second echo-magnitude image of the GRE-multi-echo image was co-registered linearly to the T2-weighted data, and the transformation matrix (QSM-T2) was used to co-register the QSM to T2-weighted using flirt. The QSM-T2 transformation matrix was then inverted to co-register the ROI masks, generated on the T2, to the high resolution QSM data. All registered ROI masks were then used to extract specific white matter frequency shift and susceptibility values. For frequency shift data, see Supplementary Material. QSM images from the interaural regions 3.5, 3, 2.5, 2, and 1.5 mm are shown in **Figure 3A** (51).

#### Immunofluorescence and Immunohistochemistry

All animals were perfused immediately after the MRI scan using 1% sodium nitrite in 0.1 M PBS followed by 4% paraformaldehyde (PFA). Brains were removed and first stored in 4% PFA for 16 h in the fridge and then into phosphate buffer saline (PBS) with sodium azide, after which brains were then pre-processed for paraffin sectioning and sectioned coronally at 10 μm (57). Immunofluorescence was performed to visualize myelin basic protein (MBP). Two brain sections which included the regions of interest between bregma levels −0.8, and −2.0 mm (selected to match with regions where we noted MRI changes) per animal from each time point were selected and immune-labeled with primary antibody for anti-myelin basic protein (MBP). Sections were first dewaxed and then treated with Revealit (ImmunoSolution, Brisbane, QLD, Australia) antigen recovery solution for antigen recovery. Sections were then washed with PBS and incubated with blocking buffer for 30 min before primary antibody incubation (1:250; rat-monoclonal anti- MBP, Sigma, M9434) over three nights at room temperature. After washing with PBS, sections were then incubated overnight in secondary antibody Alexa Fluro 568 (1:1,000) at room temperature. Sections were washed again and incubated with 4′,6-diamidino-2-phenylindole (DAPI; 1:5,000) for 2 min followed by a saline wash and cover-slipped with vectoshield (Vector Laboratories) mounting media. Stained slides were then observed and imaged at 20 × resolution using an upright fluorescent microscope using the Zeiss Axio Imager by Carl Zeiss. 20 × high-resolution images were then opened in IMRAIS image analysis software (Bitplane; version 9.2). The Allen mouse brain atlas and AMBMC atlas was used as a reference to select crop the regions of interest i.e., R1, R2, and R3. For consistency, cropped regions were scored visually by two blinded investigators. Score 0 was given to regions with hardly detectable MBP immunoreactivity, 1 was given for mild, 2 for moderate, and 3 to the regions with strong immunoreactivity strength Inter-observer variability rates were calculated as the percentage difference in the scores (−1.25, 1.8, and 2.8% for R1, R2, and R3) and were deemed satisfactorily minor and the median scores per animal andgroup were calculated. Images from random animals were chosen from each group to represent the staining from bregma region −2 mm shown in **Figure 4**. The whole-brain greyscale image shown in **Figure 4** was generated by using a threshold value of 9.

Further, immunohistochemistry was performed to quantify astrogliosis in white matter. Four sections per animal were chosen from bregma levels −0.8, −2, 2.5, and 3.5 mm and immunostained for glial fibrillary acidic protein (GFAP) following the protocol described in our previous study (57). Slides were scanned at 20 × resolution using an Aperio AT2 slide scanner from Leica Biosystems. Regions of interest were then cropped for each animal using the Allen mouse brain atlas and AMBMC atlas as a reference. Cropped regions were scored semi-quantitatively for GFAP staining intensity on a 0–3 scale by a blinded investigator. Score 0 was given normal-appearing regions or the regions with hardly detectable staining, a score of 1 was given to mild, 2 to moderate, and 3 to regions with the strongest intensity of GFAP immunoreactivity, respectively. Median scores across all section per animal were calculated and plotted for sham and TBI animals per group. Images from bregma region −2 mm were shown in **Figure 5**.

### Statistics

Prior to fitting the statistical models, we examined the normality of all the data using Shapiro–Wilk tests. Skeletonized DTI maps of TBI groups were tested for voxel-based statistical differences against the sham group by using an unpaired *t*-test performed using a non-parametric permutation test with 5,000 permutations (FSL-randomize). The results were corrected for multiple comparisons by using threshold-free cluster enhancement (TFCE), and the fully corrected *p* < 0.05 was considered significant. For the ROI analyses, two-tailed unpaired *t*-tests using *p* < 0.05 (*z* = 1.96) were applied to assess changes in axial diffusivity, radial diffusivity, susceptibility, and frequency shift. All the data were analyzed and plotted using Graph Pad Prism 7 (GraphPad Software, San Diego, CA).

For GFAP and MBP, the median and interquartile range of the median scores were plotted for the TBI and sham group using GraphPad Prism 7. Kruskal Wallis tests were applied for analyzing changes in GFAP and MBP signals followed by Dunn's multiple comparisons *post hoc* tests. Corrected *p*-values are reported. For correlations, Spearman correlations were performed, and *r*^2^ and corrected *p* values are reported.

## Results

### Temporal Changes in Axial and Radial Diffusivity Post-injury

Voxel-based statistically significant differences between the TBI and the sham groups for DTI measures AxD and RD are shown in Figure 1. At the 5 h post-injury time point, a significant increase was observed in RD near the injury (R1 and R2) while no significant difference was noted in AxD. The initial increase in RD started to significantly decrease in focal R2 region on day 1, while no significant changes were noted in R1 and R3. By day 3, more pronounced decreases in RD and AxD were present (see R2 near the injury and slightly diffused changes around R1 and R3). A decrease in AxD values persisted until day 7 and 14, but was evident only in the R1 and appeared to revert partially by 30 days post-injury. The decreased RD values observed on day 3 in R1 of the TBI group vs. the sham group were reversed by day 7 and persisted for day 14 and day 30 post-injury. At the injury site, RD values were significantly higher than the sham group on day 7, 14 and 30, specifically in the region R2 and internal capsule.

In the region of interest-based analysis, we observed a significant decrease in AxD in R1 and R2 after 3 days in the TBI group as compared to sham group (*p* < 0.02). On day 7, a significantly decrease in AxD was only observed in R2 compared with the sham group (*p* = 0.01). In radial diffusivity graphs similar to voxel-based results we observed a significant increase in RD in R1 5 h post-injury compared to the sham group (*p* = 0.02). Although we did not see any significant change in RD at other time points in region-based analysis the trend of decrease in RD at day 3 followed by further increase was similar to that of our voxel-based results, indicating that TBSS is a more sensitive detection method than region-based analysis.

### Tractography

**Figure 2:**
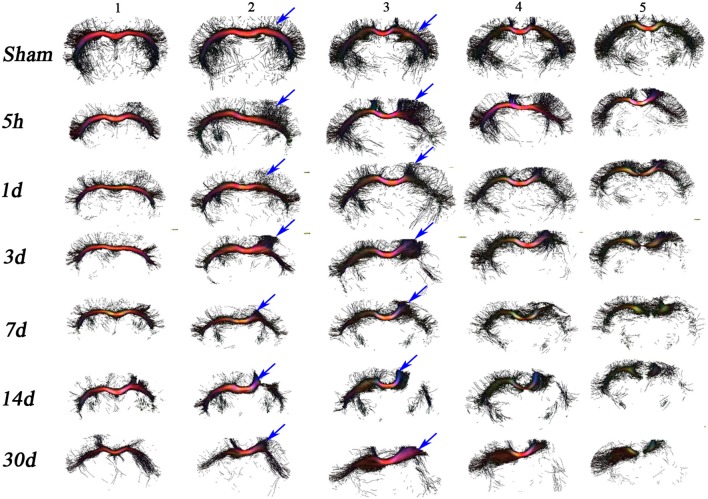
*In vivo* DTI tractography. Images depicting fiber tracks for a representative mouse from the sham and TBI groups at each time point have been provided to help understand the changes in the structure and directionality of water diffusion in white matter. Seed masks for corpus callosum were applied to generate tracks demonstrating the origin of additional tracts from the center of the CC (corpus callosum) region toward cortex in the TBI groups vs. sham group. After day 3, 7, and 14 post injury, tracks in the ipsilateral region were almost gone, loss of tracks was visible in the contralateral side as well. At day 30 similar loss of tracks on the ipsilateral and contralateral was seen.

In addition to the DTI changes, we also provide a representation of white matter seeded fiber tracks in the sham and TBI mice at different times after injury in Figure 2. Fiber tract locations are inferred from the direction of water diffusion in the tissue, and injury causes localized damage and potentially more free diffusion (i.e., physical breaking of tissue followed by inflammatory processes, as shown in **Figure 5**). Therefore, an increase in tracts at the injury site highlighted by the arrows in the second and third columns in Figure 2 should not be interpreted as a formation of new tracts. Instead, this region should be treated as a region where tractography is essentially inaccurate. However, excluding the injury site, it is of interest and possible to interpret the reshaping of tracts across the entire white matter in the ipsilateral and contralateral side over time.

The changes in comparison to sham suggest that the injury impacts white matter fibers in two ways. First, tract projections into the mouse cortex decrease overtime after injury (see for example columns 1–3) in the contralateral and ipsilateral hemispheres. Second, the damage to the ipsilateral white matter appears greater than the damage to the contralateral side, as can be seen in columns 3–5 especially at later time points. These results suggest progressing axonal damage and/or structural impairments, since ipsi- and contra-lateral projections into the mouse cortex are mostly lost over time.

### Quantitative Susceptibility Changes in the White Matter Post-injury

**Figure 3:**
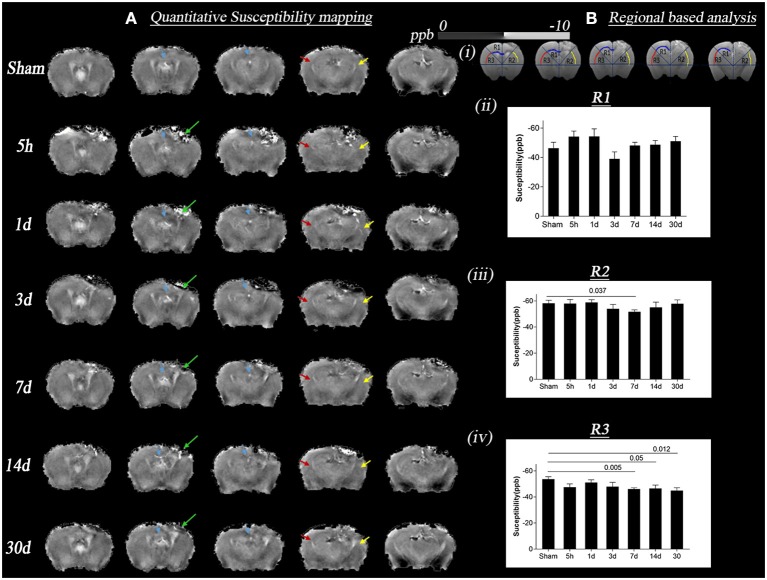
Change in quantitative susceptibility in sham and TBI group at different time points (5 h, 1 day, 3, 7, 14, and 30 days) post-injury. **(A)** Represents *in vivo* mean susceptibility maps of sham and TBI groups at different time points derived from quantitative susceptibility phase data. The green arrow marks the lesion areas, which are observed through contrast changes. Sections refer to the interaural regions at 3.5, 3, 2.5, 2, and 1.5 mm. **(B)** (i) shows the regions of interest (ROI) R1, R2, R3, respectively, marked by yellow, red, and blue arrows on the susceptibility maps, the areas where changes in DTI voxel-based analysis were seen. T ipsilateral and contralateral corpus callosum and external capsule were picked as the areas of interest for ROI analysis; **(B)** (ii) represents the graph with *p*-values for susceptibility changes in R1, the maximum increase in susceptibility was on day 3 TBI vs. sham groups, however not significant; **(B)** (iii–iv) represents the graph with *p*-values for susceptibility changes in R2/ipsilateral, and R3/contralateral. The maximum increase in quantitative susceptibility in both the regions was observed on day 7 TBI vs. sham groups. A more significant change was noted in the R3 region. Values were expressed as mean ± SEM and non-parametric student *t*-tests were performed and corrected *p*-values were reported. Sections refer to the interaural regions 3.5, 3, 2.5, 2, and 1.5 mm as per AMBMC mouse brain atlas (51).

Figure 3 summarizes bulk magnetic susceptibility of tissue findings in sham and TBI mice. Figure 3A depicts the group-mean susceptibility maps at all time points. Figure 3B (i) shows the region based analysis of the three regions R1, R2, and R3 selected as per DTI results (R1-region corresponding to the injury site in the contralateral hemisphere, R2, and R3-corresponding regions in ipsilateral and contralateral hemispheres away from injury site). Figure 3B (ii), (iii), and (iv) shows the statistical differences in susceptibility in R1, R2, and R3 regions of TBI and sham groups. A significant increase in susceptibility was observed in R2 in the day 7 TBI group when compared with the sham group (*p* = 0.037). In R3, susceptibility values were significantly increased in days 7, 14, and 30 TBI groups as compared to sham group (*p* ≤ 0.012). Notably, susceptibility maps near the injury site (R1 and R2) are influenced not only by myelin but also iron, and they are known to co-localize in tissue. Both iron and myelin are known to have vastly different magnetic susceptibilities. As such, susceptibility maps reflect the combined contributions from myelin and iron, and cannot be interpreted as pure changes in myelin content.

### Myelin Basic Protein (MBP) Changes in White Matter Post-injury

**Figure 4:**
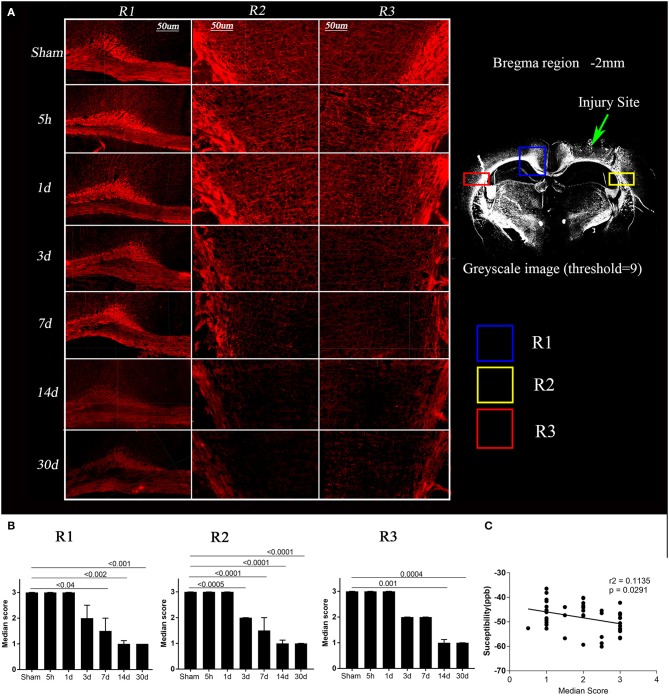
Loss of myelin basic protein (MBP) expression post-injury and its correlation with susceptibility. **(A)** Images depicting regions R1, R2, and R3 for a representative mouse from the sham and TBI groups at each time point are shown, red = MBP (in left). A 20 × greyscale image from a TBI animal (in right) from bregma region −2 mm has been shown to highlight the regions R1, R2, R3. Whole brain images were opened in IMARIS and were zoomed in to the scale bar = 50 μm to crop the regions of interest. **(B)** Semi-quantitative analysis results for MBP immunoreactivity were plotted, indicating a significant loss of MBP binding over time in day 3, 7, 14, and 30 TBI groups as compared to the sham group. Kruskal–Wallis tests with Dunn's multiple comparisons post tests were performed. Median and interquartile range were plotted with corrected *p*-values. **(C)** In R3, loss of MBP immunoreactivity was significantly correlated with increased quantitative susceptibility values, Pearson Correlation.

**Figure 5:**
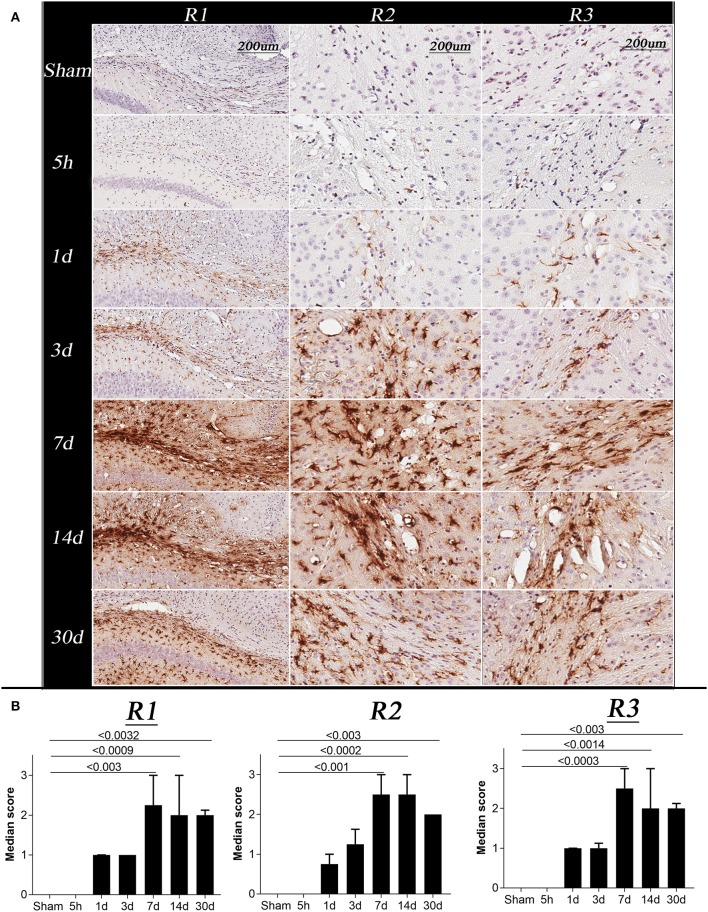
Changes in glial fibrillary acidic protein (GFAP) immunoreactivity in sham and TBI mice at specific post-injury time points (5 h, 1 day, 3, 7, 14, and 30 days). **(A)** R1, R2, and R3 of representative animals for the sham and TBI groups, showing changes in GFAP immunoreactivity following injury, all images were acquired at 20 × resolution; Scale bar = 200 μm. **(B)** Represents the graph for the astrogliosis median score, Kruskal–Wallis tests with Dunn's multiple comparisons post tests were performed. Median and interquartile range were plotted with corrected *p*-values shown on the graphs. The overall score of sham and 5 h in all the regions were 0. A significant increase in GFAP immunoreactivity was observed on day 1, 3, 7, 14, and 30 in all the regions with a maximum change on day 7 post injury.

Figure 4 shows the changes in myelin binding protein—immunoreactivity in white matter tracks after TBI. The MBP immunoreactivity scores were significantly different between time points in all 3 regions (Figure 4B, Kruskal Wallis test *p* < 0.0001). While at 5 h and 1 day, no semi-quantitative changes were observed in MBP signal (Figure 5B, *p* > 0.9), the loss of MBP signal was evident in R2 by day 3 (*p* = 0.0005). On day 7, the MBP signal associated with white matter fibers projecting into the cortex started to fade significantly in R1 (*p* < 0.05), R2 (*p* < 0.0001), and non-significantly in R3 (*p* = 0.07) in the TBI group compared to the sham group that prevailed significantly up to day 14 (*p* < 0.002) and day 30 (*p* < 0.001), indicating post-injury myelin loss. By performing correlations we observed that loss of MBP signal in R3 was slightly correlating with susceptibility increase (*r*^2^ = 0.1, *p* = 0.02).

### Changes in Glial Fibrillary Acidic (GFAP) Immunoreactivity Post-injury

The GFAP immunoreactivity scores were significantly different between time points in all 3 regions (Figure 5B, Kruskal Wallis test *p* < 0.0001). In R1, R2, and R3, GFAP immunoreactivity was barely detectable in the sham and 5 h TBI group (Figure 5B, *p* > 0.9). On day 1 and 3, a mild increase in GFAP immunoreactivity was observed in all three regions of TBI group, however, the increase was non-significant (*p* > 0.3–0.9) as compared to sham group. The strongest intensities of GFAP staining in R1, R2, and R3 were seen on days 7 and 14 compared to the sham group (*p* < 0.002). A significant increase in GFAP immunoreactivity was still seen at 30 days post-injury (*p* < 0.003).

## Discussion

This work features QSM as a sensitive technique that can specifically measure the subtle temporal dynamics in white matter myelination following injury and emphasizes the precedence of combining QSM and DTI to discern discrete facets of the time-dependent injury-triggered microstructural alterations in the rodent brain. In the controlled cortical impact mouse model, QSM was associated with significant temporal changes in white matter contralateral to the injury, representative of demyelination-remyelination modeling processes, while DTI measures appeared to reflect multiple pathologies such as axonal swelling or injury and myelin damage. The findings of this study are suggestive of the specificity of each technique and their conjoint potential in TBI.

### Oedema or Axonal Swelling Early After 5 h to Day 1 Post-injury

The controlled cortical impact model is a well-established open head injury model in rodents (58, 59). Injury has been characterized by loss of cell integrity, oedema, neuronal damage, axonal injury, metabolic stress, ischaemia, axonal swelling, axonal tearing and shearing, demyelination, and neuronal cell death (60–63). Fractional anisotropy, a DTI derived metric, has been widely used clinically and preclinically for the measurement of microstructural changes in white matter following different types of brain injuries (11, 15, 16). However, the measurement has been shown to lack specificity with respect to pathology (17, 18). Other DTI-derived measures, namely axial and radial diffusivity, have been shown to provide more robust information on axon and myelin integrity (3, 19). Changes in AxD have been linked with axonal damage and changes in RD reflect changes in myelination, or myelin integrity (19–21). Notably, in the case of more severe injuries, AxD and RD tend to provide insufficient information on the specific white matter changes taking place (29, 30). Therefore, supporting detail needs to be derived from additional data, such as magnetic susceptibility.

The results from our TBSS analysis showed a consistent pattern of white matter microstructural changes in the corpus callosum extending to the external capsule (see Figure 1). AxD, a marker of axonal damage, did not change immediately after 5 h of the injury except at the injury site, but RD a marker of oedema and myelination did increase. Early post-injury increase in RD without a measurable change in AxD is consistent with existing human and animal studies (64, 65), wherein they associated the specific changes in AxD and RD with tissue oedema and axonal swelling, and not axonal tearing (24, 66). The increase in RD at 5 h post-injury was also similar to a human study by Perez et al., in which TBI patients were found to have increased RD in their first scan 1-day post-injury. Perez et al. linked the increase in RD with oedema followed by demyelination. Their reported increase in AxD is opposite to our findings, potentially due to different injury severities (25). We observed a similar trend in MBP signal over time following injury (see Figure 4 for images from a representative mouse), implying demyelination at later time points after injury. An increase in RD could also be due to a loss in white matter fiber anisotropy (27, 65) along with mild axonal changes (26, 67). Such post-injury pathologies may be followed by ischaemia and inflammation (Figure 5) leading to more restricted extracellular diffusion reflected by a reduction in RD and AxD as seen on day 1 (68, 69). Other factors such as hindrance to water diffusion due to the presence of cell debris and high lipid content within the area of necrosis and decreased water level in the myelin sheaths can change diffusivity in tissue in addition to microstructural changes (70). In support of our findings, Babcock et al. also reported a reduction in RD in a few white matter regions 4 days after injury in a cohort of young patients (71).

To date, limited literature on the controlled cortical impact model exists in relation to magnetic susceptibility. A study on rats in the CCI model found increased susceptibility at 2 days post-injury at the injury site as well as contralateral to the injury site in white matter which was similar to our susceptibility increase on day 1 and 3 post-injury. However, these changes were only found in the group of animals with higher radial diffusion in these areas, a finding contradictory to our TBSS results in Figure 1 (34). From the DTI results, they concluded oedema to dominate over axonal and myelin damage, followed by demyelination supported by their susceptibility findings 2 days post-injury. They did not observe any change in these measures on day 7 and 14 post-injury, whereas we observed increased susceptibility up to 30 days. Injury severity, the region of the white matter chosen in this study, and different analysis methods could be reasons for these discrepancies.

Existing results suggest that within hours post-injury, AxD and RD do not reflect axonal damage, demyelination, or astrogliosis. However, oedema and axonal swelling may stretch the myelin sheath and lead to an apparent increase in water diffusion in the direction perpendicular to the fiber bundle orientation. Changes in frequency shift may reflect changes in the myelin sheath and myelination (see Supplementary Material).

### Axonal Damage and Myelin Fragmentation

At day 3 post-injury, reduction in both AxD and RD was evident in the white matter regions focal to the injury site, i.e., R1 and R2 in Figure 1. Decreased AxD in the TBI group in comparison to the sham group was also observed around R3 (contralateral corpus callosum/external capsule). Reduction in AxD persisted for at least seven days in the TBI animals. Interestingly, day 3 AxD changes were consistent with day 4 TBI findings by Mac Donald et al., in a similar mouse model of injury, but they did not report a significant change in RD (26). In addition, their reported AxD values returned to a normal level after 7 days and RD increased. They found a correlation of their findings with amyloid precursor protein (APP) staining for axonal injury (26). Their average RD value started to increase after 3 days post-injury and remained high in the corpus callosum up to 1 month, the last time point studied. The increase in RD correlated with myelin damage, myelin loss, and gliosis in rodent TBI models (11, 26, 34). Increased RD without any noticeable change in AxD after 1-month in our study was similar to the findings of Song and colleagues in shiverer mice—demyelination, but no axonal injury or inflammation was detected in their model of retinal ischemia (19). Although the retinal ischemia is vastly different from TBI, observed changes in AxD and RD with time after ischemia were similar to our findings post-TBI. These AxD and RD changes in their study were shown to be driven by axonal and myelin damage (19). Consistent with existing literature myelin loss was also seen in our TBI animals with time (Figure 4) (27, 72) which could be the factor responsible for observed RD changes however we did not observe any significant correlation between both.

Findings from the GRE-MRI data complemented the microstructural white matter changes after 3 days post-injury. We observed an increase in susceptibility in selected regions, complemented by decreases in AxD and RD followed by AxD reversion and increase in RD. Interestingly, in R3 (see Figure 3), the region contralateral to the injury, we observed a change in susceptibility without pronounced changes in DTI measures. Li et al. found a similar increase in white matter susceptibility in the group with increased RD based on a rat CCI model (34). Furthermore, they showed a correlation between increased susceptibility, demyelination, and increased RD. In another similar longitudinal CCI-mouse study, changes in different myelin proteins, i.e., myelin basic protein (MBP)—a marker of myelination (73), 2',3'-cyclic-nucleotide 3'-phosphodiesterase (CNPase)—a marker of myelinating oligodendrocytes (74), and myelin oligodendrocyte glycoprotein (MOG)—a marker of myelin protein expressed by oligodendrocytes (75) were investigated in the corpus callosum by performing western blot (72). Changes in the expression of different proteins with time after TBI were also supporting our susceptibility results. A post-injury decrease in MBP expression bilaterally in the corpus callosum at 7 days and up to 3 months, an increase in CNPase expression at days and an increase in MOG at 6 h and 3 days were reported. Together, this study implies diffused demyelination along with post-injury activation of myelin repair pathways (72). Similarly, we showed a TBI-induced loss in MBP expression after 3 days post-injury that remained low up to our 30-day time point. Our susceptibility changes in R3 correlated with MBP loss observed over time. However, R1 and R2 shared the trend in susceptibility changes (increase and then normalization) with time, which was different from R3 (increase). As R1 and R2 were close to the injury site, the susceptibility signal generated in these regions might be affected by other factors, such as blood iron and immune responses. These pathologies together can generate mixed-signal and may lead to such discrepancies as observed between R3 and R1, R2 (76, 77). In areas with increased GFAP activity the magnetic susceptibility signature of iron and myelin changes and it is difficult to isolate myelin from iron during the pathological cascade groups (78, 79). Also, in white matter, iron deposition has been shownto be minimal compared to the known demyelination process (36). We, therefore, believe that the changes in R3 are predominantly driven by demyelination rather than iron deposition supported by myelin histology. While in R1 and R2 the susceptibility signal is mixed and might have been contaminated by iron.

Based on this evidence and our findings, we conclude that DTI metrics from day 3 post-injury onward may reflect myelin damage and/or gliosis. However, QSM measures demyelination more directly (i.e., the amount of actual myelin protein, instead of physical damage to myelin which can lead to an increase in RD) in the regions ipsilateral as well as contralateral to injury. Moderate to severe TBI produces complex pathological changes. This study was not designed to investigate to which extent DTI and QSM correlate with demyelination, axonal injury or gliosis. Rather, this study provides evidence of changes in DTI and QSM signal over time in the areas showing loss of MBP and gliosis. There are some limitations of the current study. Although it has been previously shown that iron deposition is mostly at the injury site where blood clots are predominant as compared to diffused regions (76, 77) and white matter has minimal iron deposition as compared to the known demyelination process (36), histological staining of iron would have strengthened our findings. Further, we have not performed histology for axonal injury to justify axial diffusivity changes.

### Pathology Associated With Fiber Tracking and Demyelination Post-injury

While DTI is a well-established MRI technique to detect white matter microstructure changes, fiber tracts obtained from DTI data can non-specifically change in the presence of several pathologies associated with brain injury, such as oedema, diffuse axonal damage, inflammation, demyelination, and apoptosis (2, 10, 67). We observed an increased number of tracts around the region of injury, an indication of more free water diffusion immediately after the injury. At day 3 post-injury, loss of tracts in R2 and R3 suggests axonal and myelin impairment, which is a finding consistent with changes in AxD and RD. Change in the color of tracts visible after day 7 (see Figure 2) implies changes in the direction of water diffusion, supporting the finding of decreased AxD and a slightly increased RD in R1. The result suggests myelin sheath expansion of myelin loss is followed by myelin fragmentation (see Figure 4). In comparison to our 1-month tractography findings, Budde et al. showed similar track changes in their rat CCI model after 2 months of injury alongside an increased RD in the corpus callosum. Taken together, post-injury oedema appears to be followed by myelin and axonal loss.

**Figure 6:**
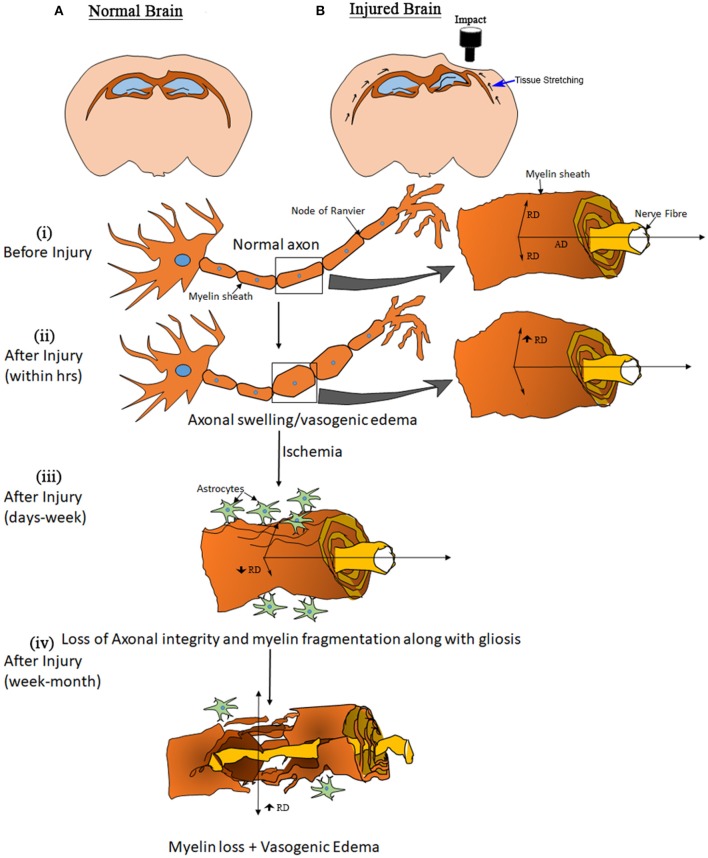
Model of elasticity. Drawings at the top show a normal brain **(A)** and the likely deformation in an injured brain. **(B)** Black arrows along the white matter in the injured brain illustrate an expected biomechanical response due to the cortical impact. The other graphics, showing a white matter fiber, elucidate the apparent processes involved after open skull injury to the brain. **(B)** (i) shows the structure of an axon representing normal myelination along with water diffusion parallel (AxD—axial diffusivity) and perpendicular (RD—radial diffusion) to the direction of fibers. **(B)** (ii) shows a swollen axon within hours post-injury, and the increased diffusivity (no change in frequency shift and QSM) may be explained by a stretching of the myelin sheath. **(B)** (iii) represents the condition of an axon within days to weeks after injury. Tissue damage causes changes that can lead to ischemia followed by inflammation, an acute immune response to clear cell debris. During this period the axons start to lose structural integrity and myelin sheath fragmentation and gliosis initiate, observed as a reduction in RD and AxD (increase in frequency shift and QSM) that may lead to myelin loss. **(B)** (iv), resulting in an eventual increase in RD (increase in QSM).

Extended on this, based on previous literature and our results, we have summarized the dynamics of the expected microstructural changes triggered following a TBI in the white matter (Figure 6). We suggest that controlled cortical impact affects the physiological and biomechanical properties of gray and white matter that can be detected using non-invasive imaging tools (80, 81). In the step (i) and (ii) of Figure 6, we demonstrated that the axons that were normal before injury appear to be swollen within 5 h post-injury that has stretched the myelin sheath reflected by increase the radial diffusivity (perpendicular) without any change in the axial (parallel) diffusivity (82) (Figure 1). This appeared to be followed by ischemia and inflammation within days leading to the shrinkage and tearing of the axonal bundles (83, 84) leading to the reduction of both axial and radial diffusivity (3, 68, 69) noticed at 3 days post-injury in our study (Figure 2). These pathologies further progress to myelin fragmentation/loss inflammation that may further increase radial diffusivity susceptibility (see Figures 1, 3 for RD and susceptibility increase and Figures 4, 5 for myelin loss and gliosis. Further, in order to understand the hemispheric variability in the MRI results, specifically the susceptibility change in focal and diffused region, we suggest that the physical impact on gray matter, specifically the white matter residing below the gray matter at the impact site, leads to increased tension on the white matter tracts projecting into both ipsilateral and contralateral hemispheres (see Figure 2). We consider this as stretching of an elastic tissue (i.e., white matter), which is attached to a relatively isotropic medium (i.e., gray matter), and shearing occurs along the white matter fiber bundles during impact (shown by arrows in Figure 6). The extent of damage depends on the impact, shearing along the gray matter, and stretching of the white matter, which might likely be greater in the regions that are closer to the impact site, i.e., ipsilateral hemisphere near the injury. This is revealed by altered DTI measures near the injury (see Figure 1) and the distinct differences in susceptibility values close to (R1 and R2) and away from (R3) the site of injury (see Figure 3). This altered white matter would then progress to myelin loss and axonal damage (see Figure 3A for GRE-MRI data-based evidence, and Figure 2 for changes in fiber tracts). In conclusion, we observed changes in RD, AD, and susceptibility at different time points during 1-month post-injury which support our hypothesis that combined QSM and DTI measures, i.e., AD and RD, have the potential to discern different aspects of the microstructural pathological changes after TBI. Future studies can provide more insight into the pathological cascade triggered post-TBI for MRI perspective.

## Conclusion

This study demonstrates the importance of QSM along with DTI-based measures, i.e., AxD and RD, for the characterization of white matter damage following TBI. We show that DTI and QSM when combined, can be considered as a specific and sensitive tool for the monitoring of myelin fragmentation and loss. Specifically, DTI appears to be a better tool when detecting acute and focal changes, while QSM seemed to be a better measure of diffuse and chronic changes. While DTI can detect complex post-injury pathologies such as oedema, inflammation, axonal damage, and demyelination, we showed that QSM can detect demyelination occurring in diffuse regions, distal to the injury.

## Data Availability Statement

The data and code scripts used in the preparation of this article will be available upon direct request as well as the conditions for its sharing or re-use according to the data sharing policies and guidelines from the University of Queensland and upon the institutional approval.

## Ethics Statement

The animal study was reviewed and approved by Animal Research Ethics Committee (AEC) of the University of Queensland (AEC number: QBI/SCMB/036/16/MAIC).

## Author Contributions

NS and FN have contributed to design of the study and data interpretation. NS contributed to generate the animal model, data acquisition, analysis, and drafting the manuscript. VV contributed to the generation of QSM data analysis pipeline and interpretation of results. FN and VV revised the manuscript for important intellectual content. XT and AM helped in data acquisition. KB helped with histology, analysis, and finalization of the manuscript. FN provided approval for publication of the content.

### Conflict of Interest

The authors declare that the research was conducted in the absence of any commercial or financial relationships that could be construed as a potential conflict of interest.
