# Supplementary material for: Combined Diffusion Tensor Imaging and Quantitative Susceptibility Mapping Discern Discrete Facets of White Matter Pathology Post-injury in the Rodent Brain
Source: Front Neurol. 2020 Mar 6;11:153. doi: 10.3389/fneur.2020.00153 (PMC7067826; doi:10.3389/fneur.2020.00153)
Supplement: Supplementary file 1 [file Table_1.DOCX]

Supplementary Material

# Frequency shift changes in white matter following injury

Supplementary-Figure 1 summarises fs findings in sham in and TBI mice. Supplementary-Figure 1A represents the temporal changes in the group-mean frequency shifts (fs) of sham and TBI mice. The three regions used in the frequency shift analysis are shown in Supplementary Figure 1B (i), and results have been plotted in Supplementary Figure-1B (ii) (R1—region corresponding to injury site in the contralateral hemisphere) and Supplementary-Figure 1B (iii) (R2 and R3—corresponding regions in ipsilateral and contralateral hemispheres away from injury site). The GRE-MRI phase signal changes around the injury site (contralateral region to R1) making analysis in this region difficult. Therefore, fs values at the injury site have not been analysed. Based on the manually segmented regions, a significant decrease in fs in R1 was not found until day 3, which persisted until day 7, after which mean fs values reverted to sham values. In R2, the white matter region distant and ipsilateral to the injury, a significant decrease in fs presented at 1 day, which further decreased day 3, before reverting at day 7 to similar values to those at 1 day. A significant decrease in fs in comparison to sham was not found at days 14 and 30. In R3, the region mimicking R2 in the contralateral hemisphere, fs values were decreased significantly at day 3 only, however, the basic trend across the various time points was similar.

# Supplementary figure

# Supplementary-Figure 1:


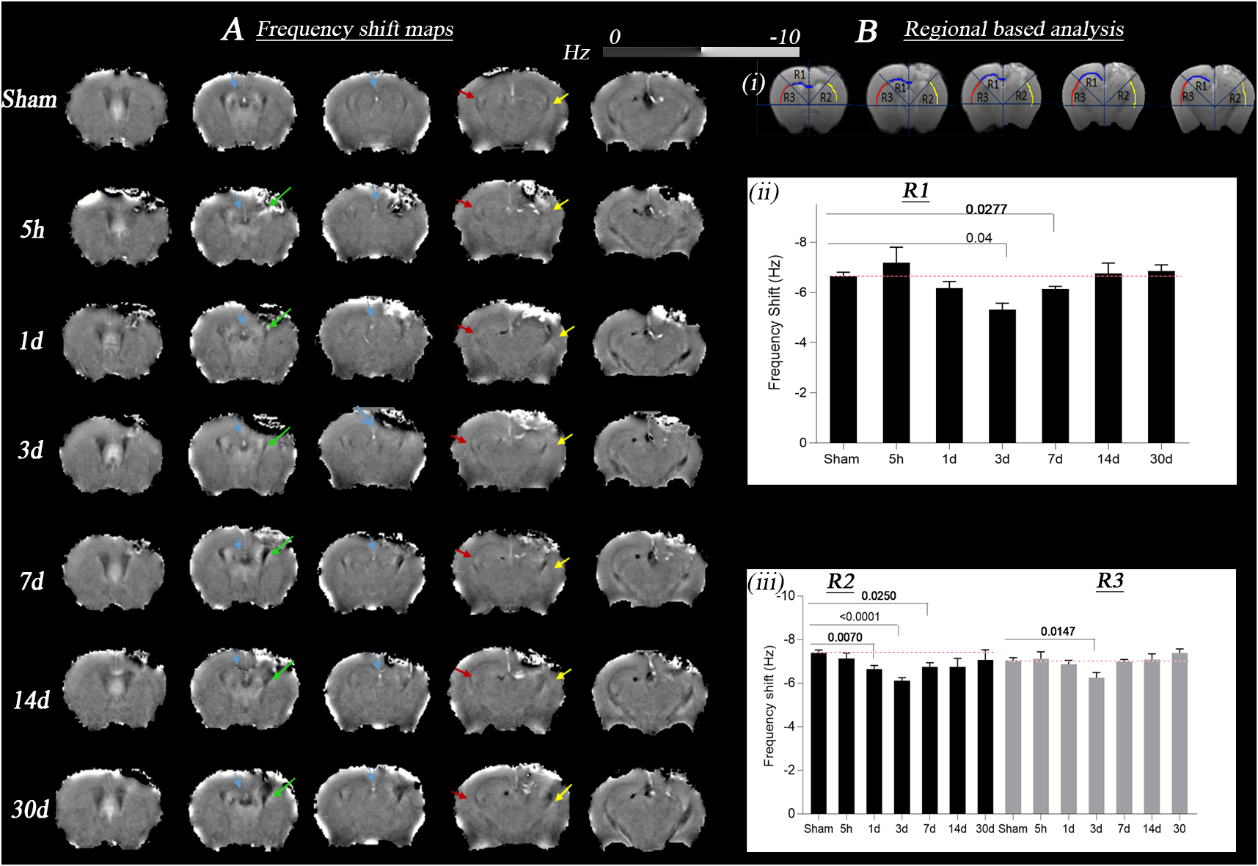


**Supplementary-Figure 1: Change in frequency shift (fs) in sham and TBI group at different time points (5 h, 1 day, 3, 7, 14, and 30 days) post-injury: A** Represents in-vivo mean frequency shift maps of sham and TBI group of different time points derived from quantitative susceptibility phase data. The green arrow marks the lesion areas which is observed through contrast changes; **B (i)** shows the regions of interest (ROI) R1, R2, R3, respectively marked by yellow, red, and blue arrows on the fs maps, the areas where we observed changes in DTI voxel-based analysis in the ipsilateral and contralateral corpus callosum and external capsule were picked as the areas of interest for ROI analysis for frequency shift data; **B (ii)** represents the graph with p-values for fs changes in R1, maximum increase in fs on day 3 TBI vs sham groups; **B (iii)** represents the graph with p-values for fs changes in R2 and R3. Maximum increase in fs in both regions was clear on day 3 TBI vs. sham groups. In R2, a significant increase in fs was also seen in one day TBI vs. sham group. Values expressed in mean ± SEM and non-parametric student t-test was performed.

# Supplementary-Figure 2:


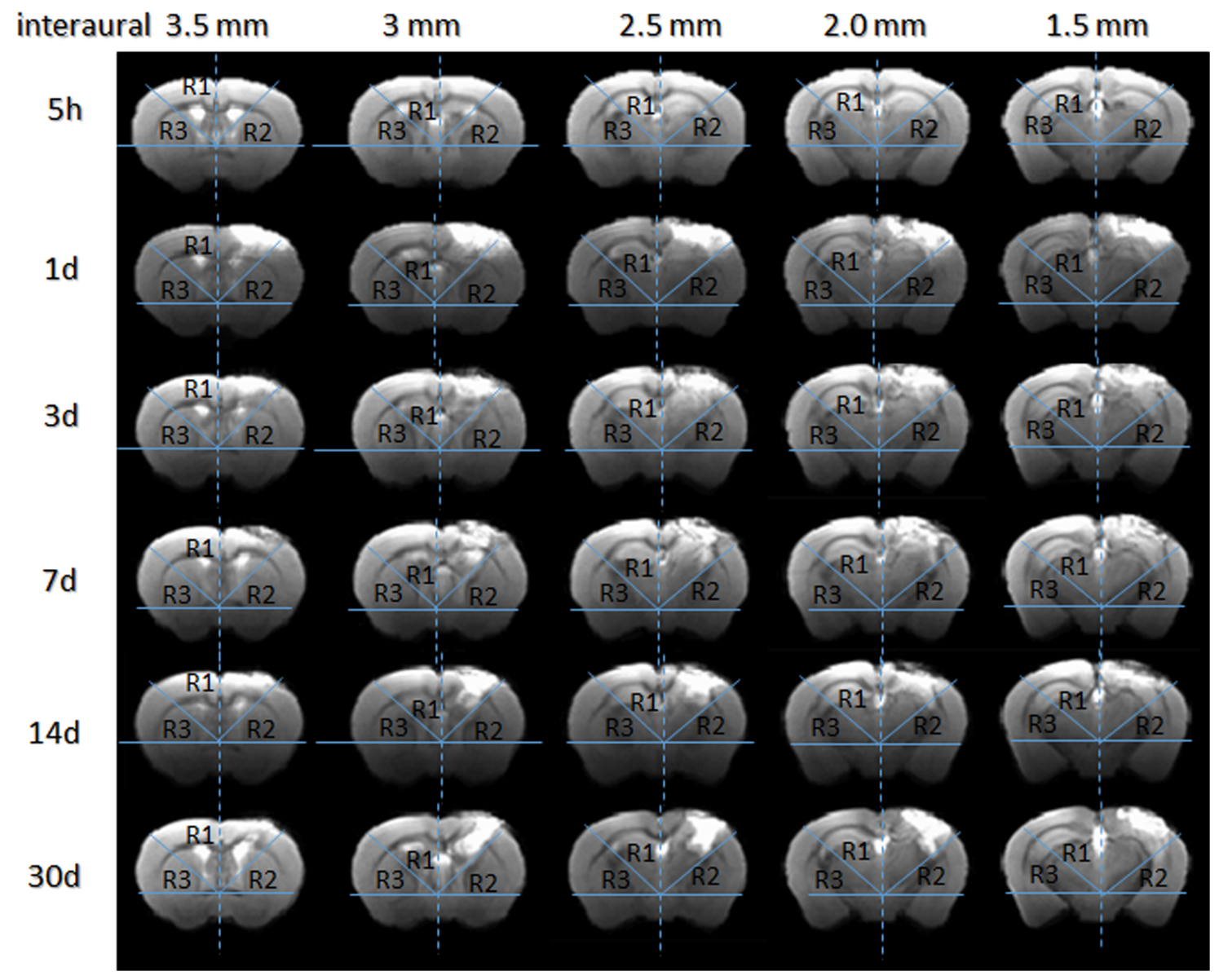


**Supplementary-Figure 2: Mean T2w images of TBI group at different time points (5 h, 1 day, 3, 7, 14, and 30 days) post-injury:** Images representing the regions of interest R1, R2, R3 for each timepoints.


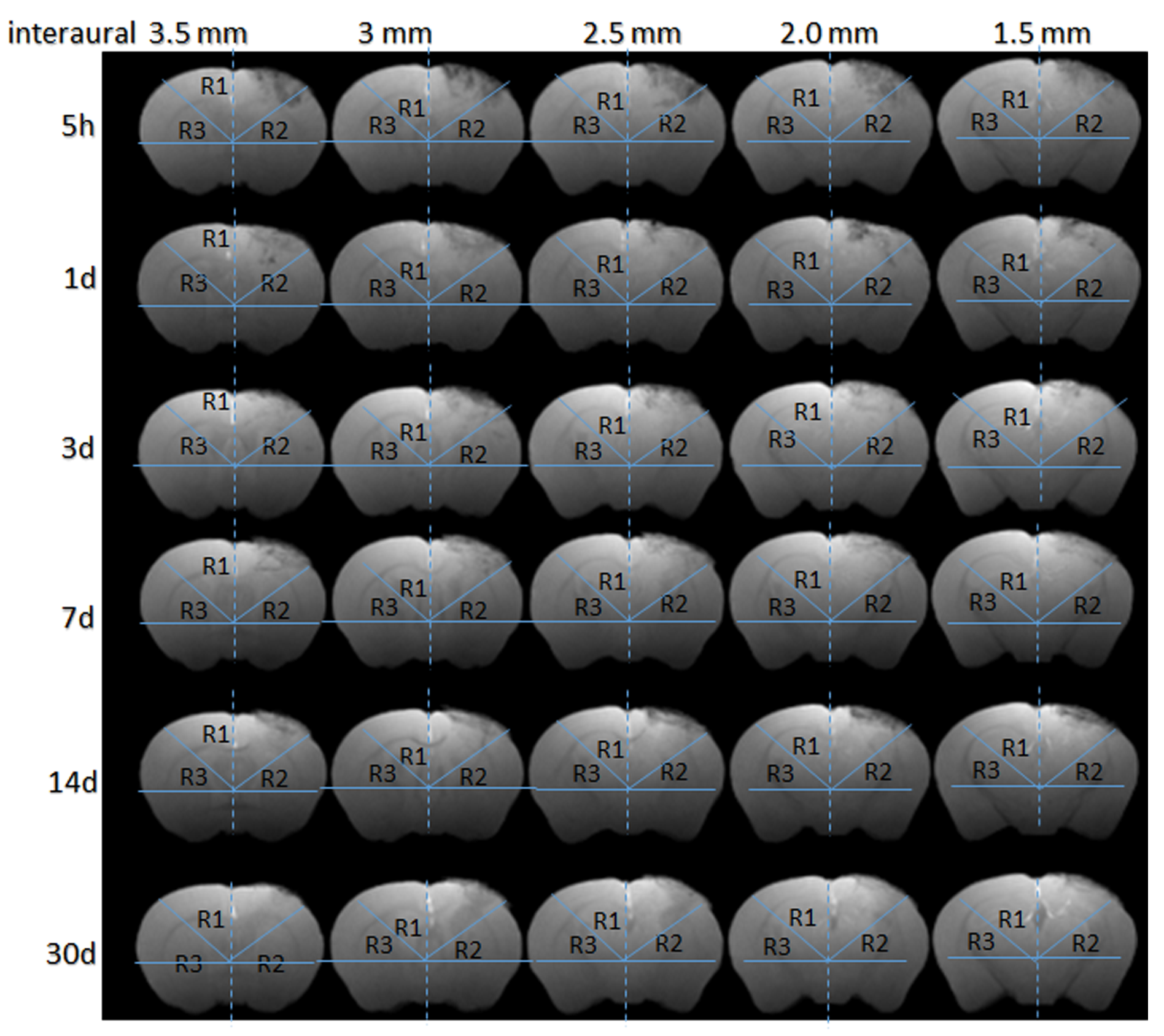


**Supplementary-Figure 3: Mean susceptibility magnitude images of TBI group at different time points (5 h, 1 day, 3, 7, 14, and 30 days) post-injury:** Images representing the regions of interest R1, R2, R3 for each timepoints.
